# Microstructure and Corrosion Resistance of Zn-Al Diffusion Layer on 45 Steel Aided by Mechanical Energy

**DOI:** 10.3390/ma12183032

**Published:** 2019-09-18

**Authors:** Jianbin Tong, Yi Liang, Shicheng Wei, Hongyi Su, Bo Wang, Yuzhong Ren, Yunlong Zhou, Zhongqi Sheng

**Affiliations:** 1 College of Mechanical Engineering and Automation, Northeastern University, Shenyang 110819, China; evantjb@163.com (J.T.);; 2 National Key Laboratory for Remanufacturing, Academy of Army Armored Forces, Beijing 100072, Chinazgysuhongyi993@163.com (H.S.); wangbobo421@163.com (B.W.); 3 Chongqing Dayou Surface Technology Co., Ltd., Chongqing 400020, China

**Keywords:** Zn-Al diffusion layer, mechanical energy aided diffusion, microstructure, corrosion resistance, electrochemistry

## Abstract

In harsh environments, the corrosion damage of steel structures and equipment is a serious threat to the operational safety of service. In this paper, a Zn-Al diffusion layer was fabricated on 45 steel by the Mechanical Energy Aided Diffusion Method (MEADM) at 450 °C. The microstructure and composition, the surface topography, and the electrochemical performance of the Zn-Al diffusion layer were analyzed before and after corrosion. The results show that the Zn-Al diffusion layer are composed of Al_2_O_3_ and Γ_1_ phase (Fe11Zn40) and δ_1_ phase (FeZn_6.67_, FeZn_8.87_, and FeZn_10.98_) Zn-Fe alloy. There is a transition zone with the thickness of about 5 μm at the interface between the Zn-Al diffusion layer and the substrate, and a carbon-rich layer exists in this zone. The full immersion test and electrochemical test show that the compact corrosion products produced by the initial corrosion of the Zn-Al diffusion layer will firmly bond to the Zn-Al diffusion layer surface and fill the crack, which plays a role in preventing corrosion of the corrosive medium and reducing the corrosion rate of the Zn-Al diffusion layer. The salt spray test reveals that the initial corrosion products of the Zn-Al diffusion layer are mainly ZnO and Zn_5_(OH)_8_Cl_2_H_2_O. New corrosion products such as ZnAl_2_O_4_, FeOCl appear at the middle corrosion stage. The corrosion product ZnAl_2_O_4_ disappears, and the corrosion products Zn(OH)_2_ and Al(OH)_3_ appear at the later corrosion stage.

## 1. Introduction

Metal materials have long been exposed to environments with high temperatures, high humidity, high salt spray, and intense sunlight, making their corrosion levels several times or even dozens of times higher than that in other environments at the same time [1,2,3,4,5,6,7,8,9,10]. In the process of metal corrosion, the mechanical properties and internal microstructure of the metal have changed. The corrosion harm includes not only the damage of internal metal structures but also the destruction of overall metal structures. Metal corrosion causes tremendous economic loss and becomes a severe threat to the development of various fields. Therefore, corrosion protection of metal materials is essential in many industrial applications [11,12,13,14]. In various environments, steel is the most used type of material in various facilities and equipment. Therefore, the corrosion protection of steel structures and equipment have flourished over recent decades [15,16,17,18,19,20].

**Figure 1:**
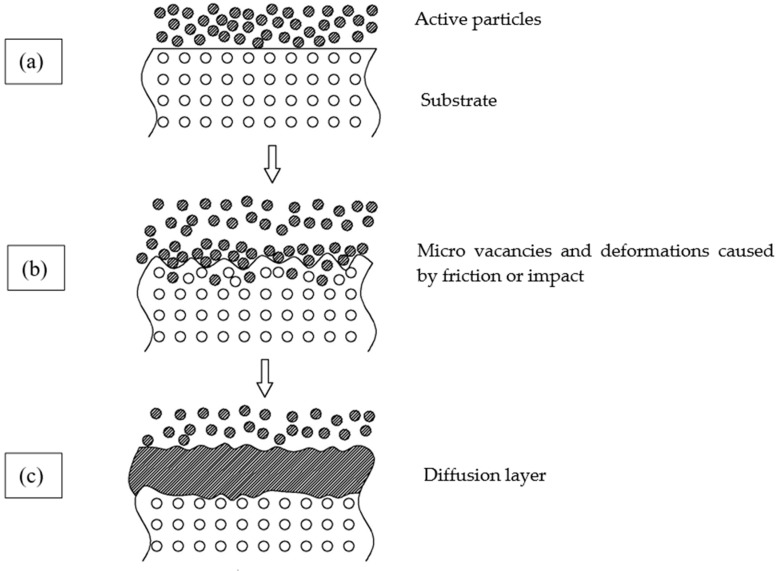
Schematic illustration of the mechanical energy aided diffusion processes.

Chemical heat treatment is usually used to improve the corrosion resistance, high-temperature oxidation resistance and hardness of metal parts [21,22,23,24]. However, due to the high temperature and long production time, it not only consumes a lot of energy, but also affects the mechanical properties of parts. Therefore, the Mechanical Energy Aided Diffusion Method (MEADM) that emerged in recent years has become an attractive metal materials anti-corrosion technology in the field of material surface strengthening. The MEADM, developed by the addition of mechanical energy (rotation, vibration, friction, etc.) in the traditional solid pack-cementation process, achieves the purpose of reducing the preparation temperature required and shortening the preparation time. As such, the MEADM develops rapidly. There are three steps in the MEADM as shown in Figure 1: (a) with the assistance of mechanical energy, the active powder particles in the diffusion agent rub and impact the surface of the heated substrate, resulting in micro vacancies and plastic deformation on the substrate surface; (b) the active particles enter the generated vacancies or adsorb into the deformed surface to form a surface solid solution or an intermetallic compound, forming an initial diffusion layer; (c) a dense protective diffusion layer is achieved by adsorbing the active particles in a continuous fashion.

Yuan et al. [25] have employed a pack-cementation process to produce aluminide coatings on both ferritic-martensitic (RAFM) and austenitic (316L) alloys at the temperature of 600–800 °C. Lee [26] has applied a pack-cementation process to form aluminide coatings at 850 °C for 2 h. However, Wang et al. [27,28] studied the MEADM and found that mechanical energy can improve the adhesion ability of Al powder on the surface of the substrate, increase the chemical activity and adhesion strength of Al, and thereby significantly reduce the aluminizing temperature, which can be reduced to 500 °C [27]. The MEADM attributes to the temperature reduction for the following reasons: (a) the powder, the workpiece, and the vessel wall collide with each other in the mechanical energy aided diffusion process to form flow and heat transfer, enhance the heat conduction and thereby accelerating the diffusion rate; (b) the moving powder particles hit the surface of the workpiece to cause activation and misalignment of the surface lattice atoms, forming supersaturated vacancies and thereby reducing the diffusion temperature; (c) the movement of the powder particles accelerates the chemical reaction of the diffusion and increases the concentration of the active Al atoms; (d) the movement of the powder particles purifies the surface of the workpiece.

Zhang et al. [29] have used the MEADM to prepare the Zn diffusion layer on Q235 steel, and found that the diffusion layer, composed of FeZn_15_, FeZn_11_, FeZn_9_, and FeZn_7_ phases, has excellent resistance to high temperature oxidation and corrosion. He et al. [30] obtained Al-Zn-Cr diffusion layer on 20 steel at 600 °C by the MEADM and concluded that the Al-Zn-Cr diffusion layer, a multi-layered structure, is resistant to high temperature oxidation and corrosion. In addition to excellent corrosion resistance and high temperature oxidation resistance, the Zn-Al diffusion layer (based on the MEADM) has the characteristics of uniform thickness, high hardness, good scratch resistance, high powder recyclability, and low environmental pollution. At present, Zn layers, Al layers, and Zn-Al layers are studied widely [27,28,31,32,33].

In this paper, in order to analyze the severe corrosion problem of steel structure and equipment in harsh corrosive environments, 45 steel was used as the substrate material, and the Zn-Al diffusion layer was prepared by the MEADM at 450 °C without changing the properties of the substrate. In this paper, we investigated the microstructures and element distribution of the Zn-Al diffusion layer. The 3D topography and corrosion mechanism of the Zn-Al diffusion layer surface were studied by full immersion test with 3.5 wt. % NaCl. The surface topography and corrosion product composition of the Zn-Al diffusion layer in different salt spray corrosion periods were analyzed. The results can provide a technical basis for long-term corrosion protection of steel structures and equipment in harsh environments.

## 2. Experimental

### 2.1. Experimental Materials and Sample Preparation Process

The 45 steel was used as the substrate material, and its specific components are shown in Table 1. The sample size was 10(L) × 10(W) × 4(H) mm.

The preparation process of the Zn-Al diffusion layer is mainly divided into three parts: pre-treatment, mechanical energy aided diffusion process, and post-treatment. The oil and rust on the surface of the substrate were removed by sanding, ultrasonic cleaning, and shot blasting (0.2 mm steel shot) during the pre-treatment. The mechanical energy aided diffusion processes are as follows:

(1) Stirring: The diffusion agent was weighed with a certain pre-calculated percentage (0.02% NH_4_Cl, 0.05% Rare Earth, 49.93% Al_2_O_3_, 35% Zn, and 15% Al) and mixed uniformly with a mixer.

(2) Loading furnace: A part of the mixed agent was taken out into the rotary furnace (a special mechanical energy aided diffusion device), the workpiece was put into the furnace, then the remaining agent was put into the furnace, and the furnace lid was tightened finally.

(3) Heating and holding: The furnace was heated in the electric furnace and rotated at a constant rotational speed of 7 r/min. The timing started when the temperature reaches 450 °C. The heating was stopped when the set holding time (4 h) was reached.

(4) Cooling: The furnace was kept rotating at a constant speed (can be increased to 10 r/min). The furnace was cooled naturally to room temperature in the air.

(5) Separation: The workpiece and the powder in the furnace were separated by filtration. The post-treatment included cleaning, alcohol wiping, drying, and testing.

### 2.2. Experimental Equipment and Parameters

(1) The cross-sectional topography, composition and element distribution of the Zn-Al diffusion layer before and after corrosion were analyzed by Scanning Electron Microscope (SEM) (Nova Nano SEM50, FEI, Hillsboro, OR, US).

(2) The phase composition of the Zn-Al diffusion layer before and after corrosion was analyzed by X-ray Diffractometer (XRD) (Smartlab, Rigaku, Tokyo, Japan). The specific test conditions were Cu target (9 KW), accelerating voltage 40 kV, tube current 40 mA, scanning rate 5°/min, test angle 10°–90°, and scanning step length 0.02°.

(3) The 3D surface topography of the sample in different immersion stages was measured by 3D Laser Scanning Microscope (LEXT OLS4100, OLYMPUS, Tokyo, Japan).

(4) The samples of different corrosion stages were obtained on Salt Spray Testing Chamber (YWX-010, SHUANGKE, Beijing, China). The specific test conditions were that the corrosive medium was 5 wt. % NaCl solution, the temperature was 35 °C, and the spray method was continuous.

(5) Electrochemical impedance spectroscopy (EIS) (amplitude 5 mV, scanning frequency range 10 mHz–100 kHz) and potentiodynamic polarization (scanning speed 1 mV/s, scanning range ±250 mV of electrode potential) were tested on an Electrochemical Workstation (IM6, Zahner, Kronach, Germany).

## 3. Results and Discussion

### 3.1. Cross-Sectional Topography and Composition Analysis of the Zn-Al Diffusion Layer

**Figure 2:**
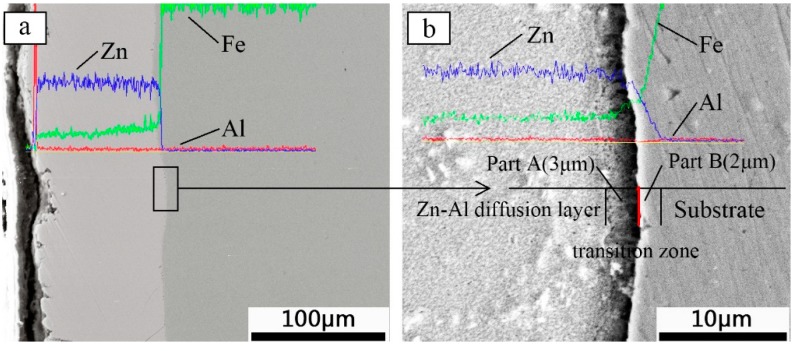

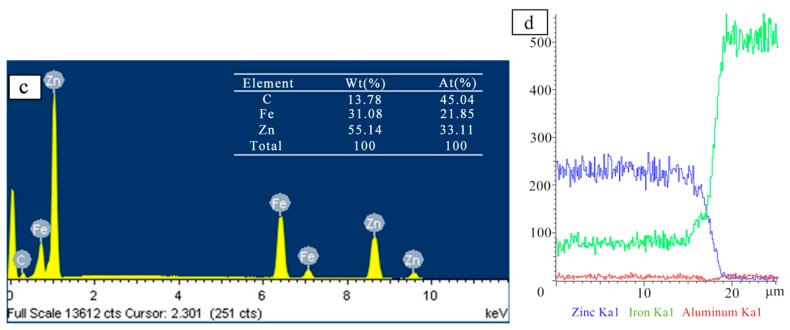
Scanning Electron Microscope (SEM) images and corresponding Energy Dispersive Spectroscopy (EDS) spectra of the Zn-Al diffusion layer in cross section: (**a**) sectional topography of the Zn-Al diffusion layer; (**b**) enlarged image of the rectangular area; (**c**,**d**) EDS spectra of the Zn-Al diffusion layer.

Figure 2 shows the SEM images and corresponding Energy Dispersive Spectroscopy (EDS) spectra of the Zn-Al diffusion layer in cross section. Figure 2b is an enlarged image of the rectangular area in Figure 2a,c,d are EDS spectra of the Zn-Al diffusion layer. It can be found that the main elements of the diffusion layer are Zn, Al, and Fe. After the Al powder is diffused by the MEADM at 450 °C, a high Al content is only detected near the diffusion layer surface, which indicates that the Al layer with a thickness of 2–4 μm is mainly present in the superficial layer. In the vertical direction of the Zn-Al diffusion layer, the content of Zn changes little, and the overall content shows a slight downward trend, whereas the content of Fe increases slowly.

On observation of Figure 2b, there is a transition zone with a thickness of 5 μm at the interface between the Zn-Al diffusion layer and the substrate. The content of Zn and Fe in the transition zone changes abruptly. The content of Zn reduces to almost 0. The Fe element content remains constant after a sharp increase. The transition zone is divided into two parts. The first part (Part A) is close to the Zn-Al diffusion layer and has a thickness of 3 μm. The other part (Part B) is close to the substrate and has a thickness of 2 μm. This part is formed by the diffusion of Zn into the substrate. A small number of pores (average diameter 0.6 μm) are found at the boundary of the transition zone by the SEM image. These pores are caused by a small amount of entrained air that cannot be discharged in time when the diffusion element permeates into the substrate. This is attributed to the fact that the ambient preparation temperature is not stable and the substrate temperature is low at the initial stage of the Zn-Al diffusion layer growth.

**Figure 3:**
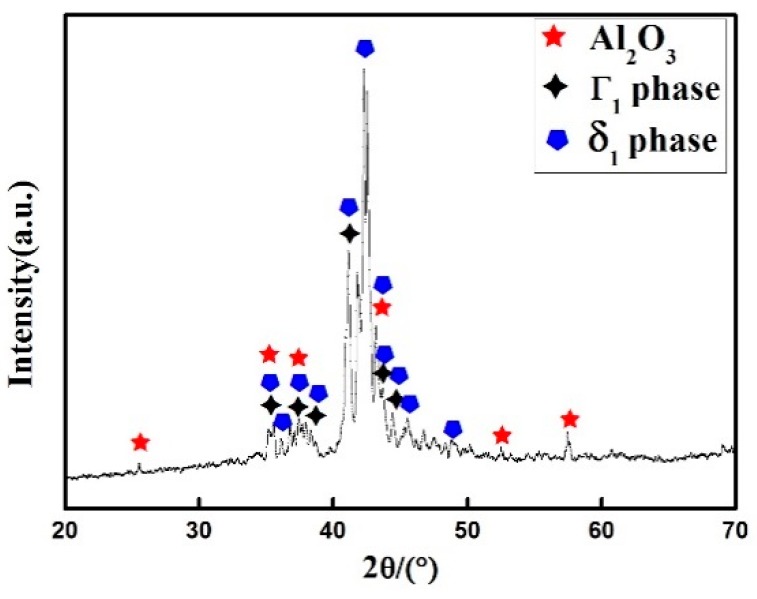
XRD patterns of the Zn-Al diffusion layer.

**Figure 4:**
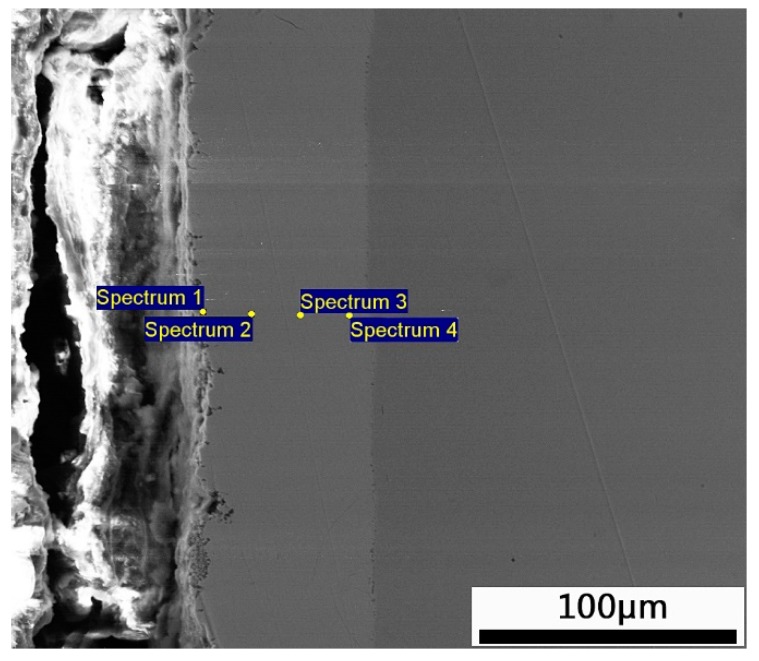
EDS spot scanning position distribution of the Zn-Al diffusion layer in cross section.

Figure 3 shows the XRD patterns of the Zn-Al diffusion layer. It is found that Al_2_O_3_ and Zn-Fe alloys of Γ_1_ phase (Fe_11_Zn_40_) and δ_1_ phase (FeZn_6.67_, FeZn8_.87_, FeZn_10.98_) are mainly formed in the Zn-Al diffusion layer. Combined with SEM images and EDS spectra, the element ratios at positions 1, 2, 3, and 4 in Figure 4, are shown in Table 2. The Zn-Fe content ratios (W_Zn_:W_Fe_) are 10, 8.2, 8.4, and 6.4, respectively. According to XRD patterns, the Zn-Fe alloy near the surface of the Zn-Al diffusion layer is mainly FeZn_10.98_, the Zn-Fe alloy near the boundary between the diffusion layer and the substrate is mainly FeZn_6.67_, and the Zn-Fe alloy in the middle area of the diffusion layer is mainly FeZn_8.87_.

**Figure 5:**
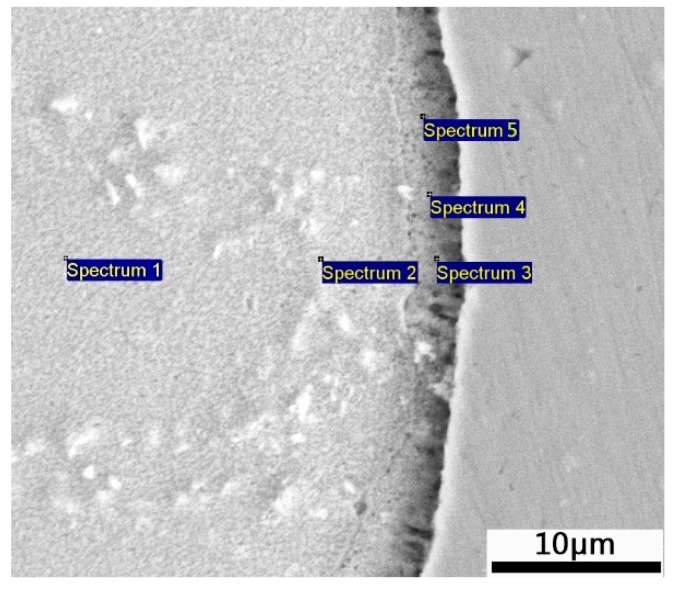
Position distribution of EDS spot scanning in the region near the interface of the Zn-Al layer.

The content and distribution of elements in the region near the interface of the Zn-Al layer were analyzed. The elements and ratios at the positions numbered by 1, 2, 3, 4, and 5 in Figure 5 are shown in Table 3. The Zn-Fe content ratios (W_Zn_:W_Fe_) in the positions numbered by 1, 2, 3, 4, and 5 are 6.8, 6.2, 5, 5.87, and 3, respectively, further indicating that the Zn-Fe alloy near the substrate is FeZn_6.67_. Analyses of SEM images and EDS spectra reveal that the closer to the substrate the Zn-Al diffusion layer is, the higher the C content is. At the positions numbered by 3, 4, and 5, the C content reaches 51.85%, 47.12%, and 47.78%, respectively, indicating that the region of the Zn-Al diffusion layer forms a carbon-rich layer near the substrate. The C content of the carbon-rich layer is much higher than that of the 45 steel substrate. This is because Zn and Al are non-carbide forming elements, which cause the C atom crowding-out effect during the formation of the diffusion layer [34]. Therefore, the diffusion layer will have a carbon-rich layer near the substrate.

### 3.2. Corrosion Resistance

#### 3.2.1. 3D Surface Topography Analysis of the Zn-Al Diffusion Layer during Full Immersion

**Figure 6:**
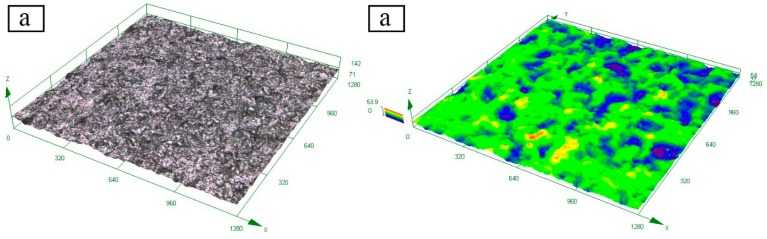

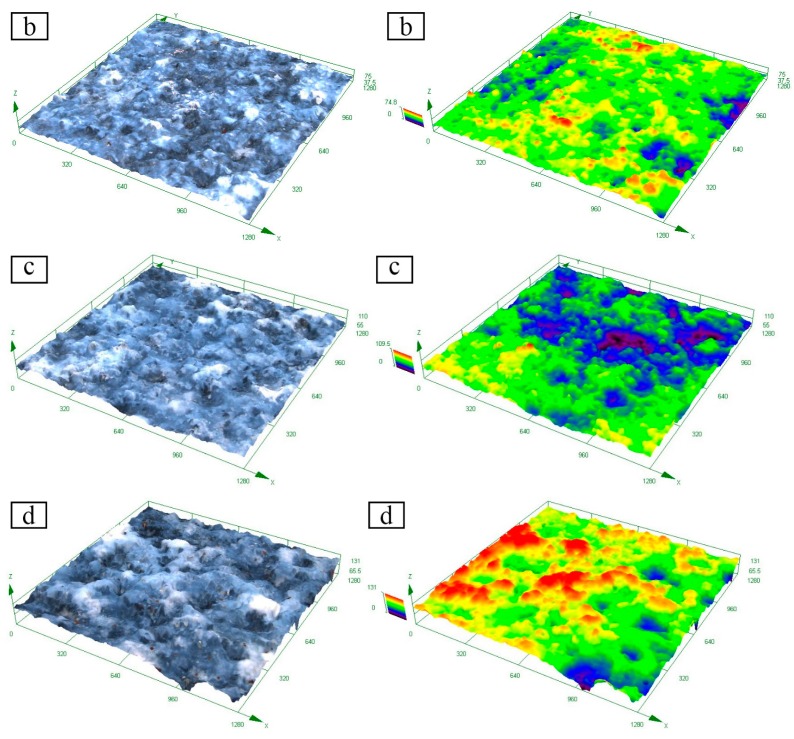
3D topography of the Zn-Al diffusion layer during full immersion: (**a**) before immersion; (**b**) immersed for 240 h; (**c**) immersed for 600 h; (**d**) immersed for 1000 h.

In order to study the corrosion resistance of the Zn-Al diffusion layer, the samples were immersed fully in 3.5 wt. % NaCl solution, and the 3D topography of the samples in different immersion stages was characterized at room temperature. The topographical images shown in Figure 6 are composed of a real graph and a color graph, and the color graph is the color mark of the height of the Zn-Al diffusion layer in different regions of the real one.

Observing the surface topography, it is found that the surface of the Zn-Al diffusion layer is uniform and has a low roughness before the full immersion test, and the drop between the high and low points is within 54 μm. The reason is that the substrate was subjected to shot blasting during pre-treatment, which caused a certain roughness on the surface of the substrate. Therefore, the Zn-Al diffusion layer is uniformly distributed on the surface of the substrate, which causes a certain degree of surface drop.

After 240 h full immersion, the surface color of the sample changed. Compared with the surface before the full immersion, more pronounced peak and pit features are exhibited, and the maximum drop between the high and low points increase to 74 μm. After full immersion, some corrosion products accumulate on the Zn-Al diffusion layer surface and form some corrosion pits, which aggravates the surface roughness. With the immersion time extended to 600 h, a large number of white corrosion products appear on the Zn-Al diffusion layer surface. As the accumulated corrosion products further increase, the area and depth of corrosion pits further increase, and the maximum drop between the high and low points reaches 110 μm. When the sample is immersed for 1000 h, the corrosion products on the Zn-Al diffusion layer surface further increase, and the surface corrosion pits are obvious. The maximum drop on the surface is increased to 131 μm and some red rust spots are observed in the real image. At this moment, a small number of corrosion pits have penetrated the entire Zn-Al diffusion layer to the substrate.

**Figure 7:**
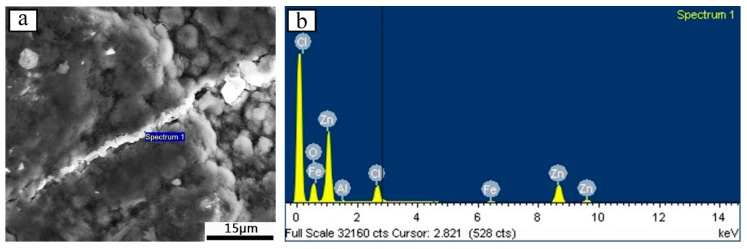
(**a**) Microstructure of the Zn-Al layer for 360 h immersion; (**b**) EDS spectra of the Zn-Al layer for 360 h immersion.

Figure 7 depicts the microscopic corrosion topography and EDS spectra of the Zn-Al diffusion layer after immersing for 360 h and ultrasonic cleaning for 10 min. The corrosion topography show that the metal powder on the Zn-Al diffusion layer surface is actively dissolved, and the flocculent corrosion products deposit on the surface, covering the entire surface. The energy spectrum analysis of the filler in the surface crack of the diffusion layer is shown in Table 4. In addition to the elements of O, Al, Fe, and Zn, the Cl element which is the main element causing corrosion is detected. It indicates that the filler in the surface crack is corrosion products produced by Cl^−^ corroding in solution. After ultrasonic cleaning, the corrosion products are still present in the crack, indicating that the corrosion products are firmly bonded to the Zn-Al diffusion layer. The firmly combined corrosion products fill the crack to help block the intrusion tunnel of the corrosive medium, which can slow down the corrosion rate of the Zn-Al diffusion layer and improve the protection ability for the substrate [35,36].

#### 3.2.2. Corrosion Behavior Analysis of the Zn-Al Diffusion Layer

In order to further study the corrosion resistance of the Zn-Al diffusion layer, a neutral salt spray test was developed. The sample was placed in a salt spray test chamber. The surface corrosion topography and the corrosion product changes of the Zn-Al diffusion layer in different corrosion stages were studied.

**Figure 8:**
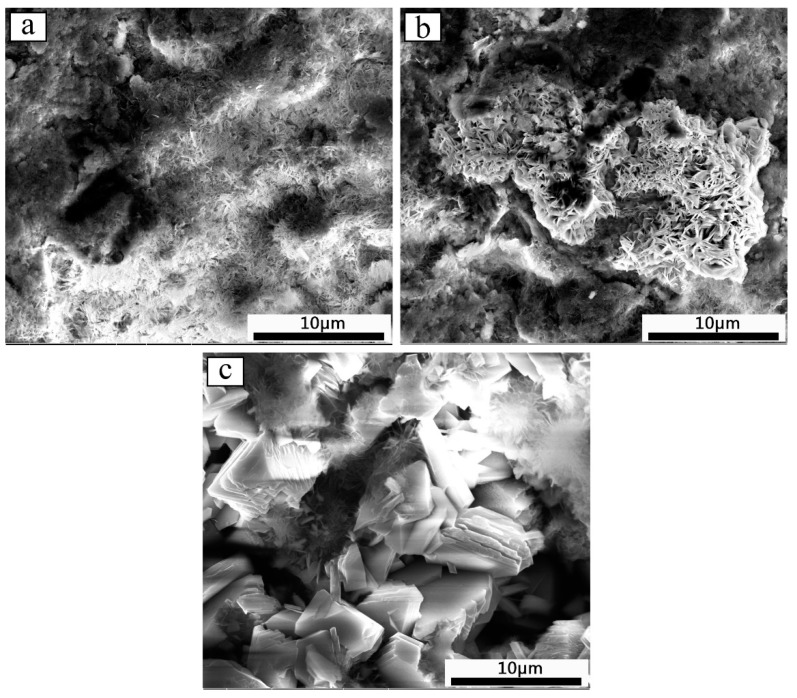
Surface topography of the Zn-Al diffusion layer during salt spray corrosion: (**a**) 168 h; (**b**) 480 h; (**c**) 1000 h.

**Figure 9:**
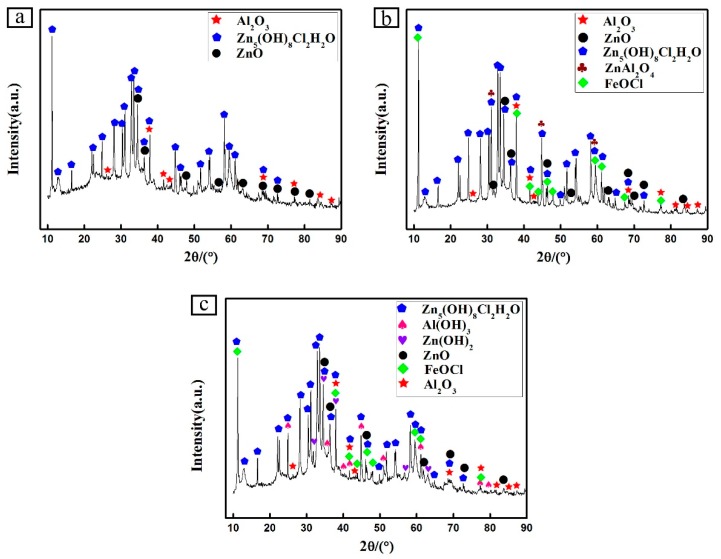
XRD patterns of the Zn-Al diffusion layer corrosion products during salt spray corrosion: (**a**) 168 h; (**b**) 480 h; (**c**) 1000 h.

The surface corrosion topography and corrosion product XRD results of the Zn-Al diffusion layer in different salt spray corrosion stages are shown in Figure 8 and Figure 9. At the initial stage of salt spray corrosion (within 168 h), a layer of flocculent corrosion products uniformly forms on the Zn-Al diffusion layer surface. The corrosion products cover the surface, so that the tunnels (the corrosion solution can invade the substrate through these tunnels) are reduced, thereby the corrosion resistance of the Zn-Al diffusion layer is improved. XRD results show that the corrosion products on the Zn-Al diffusion layer surface are mainly composed of ZnO, Al_2_O_3_, and Zn_5_(OH)_8_Cl_2_H_2_O. From the corrosion products formed, with the electrochemical reaction proceeding, Na^+^ moves toward the cathodic region, and Cl^−^ moves toward the anodic region. Zinc hydroxychloride (Zn_5_(OH)_8_Cl_2_H_2_O) and Zinc oxide (ZnO) gradually form in the anodic dissolution region.

When corroding to the middle stage of corrosion (480 h), the flocculent corrosion products on the surface have become the needle-like corrosion products that are shown by a network-like structure on the surface. According to the XRD results, it is obvious that the main corrosion products are comprised by ZnO, Al_2_O_3_, Zn_5_(OH)_8_Cl_2_H_2_O, ZnAl_2_O_4_, and FeOCl. Compared with the initial corrosion stage, the number of Zn_5_(OH)_8_Cl_2_H_2_O on the surface increases, and the density of corrosion product layer increases, which helps slow down the corrosion from corrosive medium and reduce the corrosion rate of the Zn-Al diffusion layer. In addition, the newly formed corrosion product, iron oxychloride (FeOCl), is a structurally unstable corrosion intermediate that can accelerate corrosion. However, when FeOCl releases Cl^−^, it can form FeO(OH), and the migrated OH^−^ can react with the metallic ions in the corrosive medium to develop new products. These products cover the Zn-Al diffusion layer surface, which further suppress the corrosion of the Zn-Al diffusion layer to some extent.

In the later stage of corrosion (1000 h), more agglomerated products appear on the surface of the diffusion layer. Compared with the initial stage and the middle stage of corrosion, a small number of flocculent and needle-like corrosion products distribute on the surface. The agglomerated corrosion product layer is easy to fall off. Therefore, the corrosion solution easily passes through the pores between the corrosion products and penetrates the diffusion layer. At this time, the corrosion resistance of the Zn-Al diffusion layer is weakened. XRD analyses show that ZnAl_2_O_4_ disappear on the surface and Zn(OH)_2_ and Al(OH)_3_ appear in comparison with the middle corrosion stage. The disappearance of ZnAl_2_O_4_ is due to the decrease of Al content in the diffusion layer and ZnAl_2_O_4_ formed and the shedding of ZnAl_2_O_4_ of the surface with the prolongation of corrosion time.

### 3.3. Electrochemical Performance Analysis of the Zn-Al Diffusion Layer

**Figure 10:**
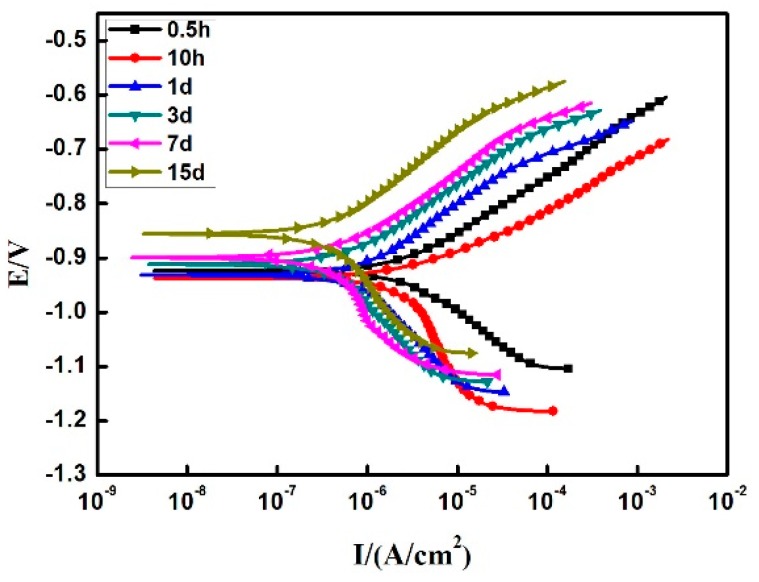
Potentiodynamic polarization curve of the Zn-Al diffusion layer.

Figure 10 depicts the potentiodynamic polarization curves of the Zn-Al diffusion layer in 3.5 wt. % NaCl solution for different immersion times, and Table 5 shows the corresponding polarization curve fitting data. Through the polarization curve analysis, it is found that as the immersion time is prolonged, the self-corrosion potential of the Zn-Al diffusion layer increases significantly, but the self-corrosion current density decreases by an order of magnitude. In other words, the corrosion rate of the Zn-Al diffusion layer decreases with the prolongation of immersion time in a certain time range.

In the whole process of full immersion corrosion, the anode Tafel slope βa of the Zn-Al diffusion layer is less than the cathode Tafel slope βc, indicating that the corrosion reaction of the Zn-Al diffusion layer is mainly controlled by the cathodic reaction. The specific reaction is as follows: the anodic reaction Zn − 2e^−^ → Zn^2+^, Al − 3e^−^ → Al^3+^; the cathodic reaction O_2_ + 2H_2_O + 4e^−^ → 4OH^−^; the total reaction 2Zn + O_2_ + 2H_2_O → 2Zn(OH)_2_, 4Al + 3O_2_ + 6H_2_O → 4Al(OH)_3_ [37]. It is found that the cathode Tafel slopes βc and the anode Tafel slopes βa are not much different during the initial immersion stage. At this time, the anodic reaction is that Zn and Al in the Zn-Al diffusion layer dissolve to produce Zn^2+^ and Al^3+^ in the corrosive medium, and the cathode absorbs oxygen to form OH^−^. With the prolongation of immersion time, the anode Tafel slope βa remains unchanged, but the cathode Tafel slope βc increases gradually. It indicates that the cathodic oxygen-absorbing reaction (the formation of the corrosion products layer) controls the corrosion reaction of the Zn-Al diffusion layer with the βc value increases. The accumulating rate of electrons in the cathode region accelerates, resulting in a decrease of the self-corrosion potential difference and the corrosion current density between anode and cathode. In addition, the polarization resistance Rp also increases greatly with the prolongation of immersion time, indicating that the corrosion products formed on the Zn-Al diffusion layer surface accumulate gradually and the corrosion rate of the Zn-Al diffusion layer decreases. The compact corrosion products formed adhere to the surface, which acts as a protective layer and slows the corrosion rate. The results of the polarization potential test are also consistent with the surface analysis results of the full immersion test.

**Figure 11:**
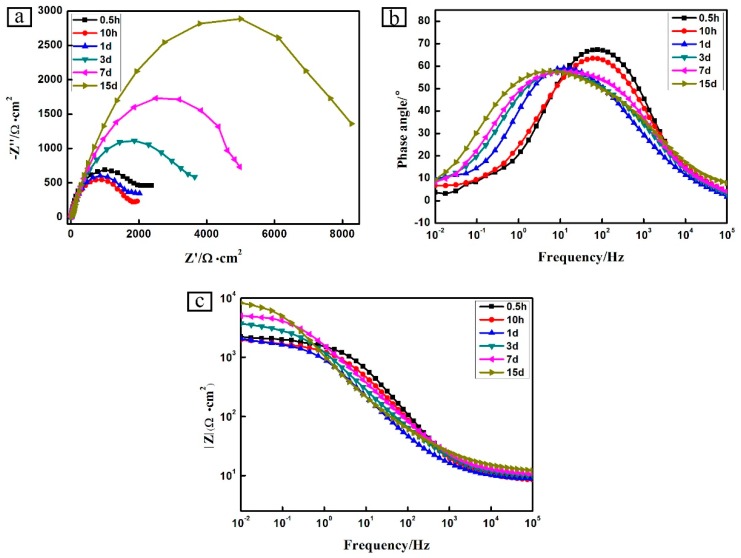
EIS spectra plots of different immersion time of the Zn-Al diffusion layer: (**a**) Bode impedance modulus; (**b**) Bode phase angle; (**c**) Nyquist.

Figure 11 presents the EIS of the Zn-Al diffusion layer in different immersion time, and Table 6 shows the impedance modulus in different immersion times. The impedance modulus diagram (Figure 11a) shows that the impedance modulus of the low-frequency region decreases within 24 h of immersion. When the immersion time is 72–360 h, the impedance modulus of the low-frequency region increases sharply, indicating that the corrosion rate of the Zn-Al diffusion layer decreases at this time. The phase angle diagram (Figure 11b) shows that only a time constant characteristic appears in the Zn-Al diffusion layer, and the peaks in the phase angle diagram gradually become higher as the immersion time is prolonged. It is concluded that the prolongation of immersion time (more than 24 h) makes the Zn-Al diffusion layer to be an isolating layer with high resistance and low capacitance, which plays a protective role for the Zn-Al diffusion layer. In the Nyquist diagram (Figure 11c), there is only one capacitive reactance arc in the Zn-Al diffusion layer, and the radius of the capacitive reactance arc decreases firstly and then increases with the prolongation of the immersion time, which is consistent with the change law of the corrosion resistance of the Zn-Al diffusion layer in the impedance modulus diagram and phase angle diagram.

The corrosion of the Zn-Al diffusion layer is a controlled process in which the electrochemical reaction gradually changes to the diffusion of corrosive medium or corrosion products. During the initial immersion stage, the impedance modulus of the low-frequency region in the 3.5 wt. % NaCl solution is low. The reactions on the Zn-Al diffusion layer surface are mainly zinc oxide, aluminum oxide, and zinc aluminum active dissolution. When the immersion time is 10 and 24 h, the impedance modulus of the low-frequency region decreases. The prolongation of the immersion time makes the electrolyte solution continuously penetrate the Zn-Al diffusion layer, causing continuous corrosion damage of the Zn-Al diffusion layer and the decreases of the impedance modulus. Therefore, as the immersion time is prolonged, the impedance modulus of the low-frequency region decreases. However, as the immersion time continues to increase, the corrosion products continuously deposit on the surface and fill into the crack. The tunnel from the corrosive medium to the substrate reduces, slowing the penetration rate of the corrosive electrolyte. Therefore, the impedance modulus of the Zn-Al diffusion layer rises rapidly in the 3.5 wt. % NaCl solution, and the corrosion rate decreases remarkably.

**Figure 12:**
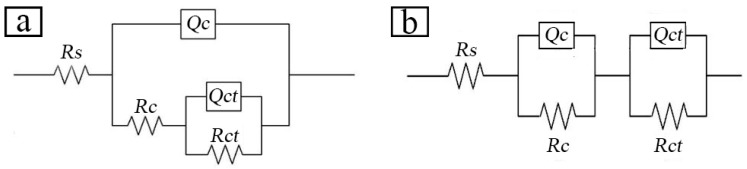
EIS equivalent circuit models of the Zn-Al diffusion layer in different immersion times: (**a**) for fitting the diffusion layer impedance data within 24 h of immersion(Rs(Qc(Rc(Qct Rct)))); (**b**) for fitting the diffusion layer impedance data after immersing for 24 h (Rs(QcRc)(Qct Rct)).

Two equivalent electrical circuits shown in Figure 12 were utilized to fit the EIS data and account for the corrosion behavior of the Zn-Al diffusion layer. The first equivalent circuit (Rs(Qc(Rc(QctRct)))) was used to fit the EIS data displaying the Zn-Al diffusion layer within 24 h of immersion, whereas the second one (Rs(QcRc)(QctRct)) was used for the EIS data displaying the impedance after immersing for 24 h. In Figure 12, Rs is the solution resistance, Rc is the Zn-Al diffusion layer resistance, Rct is the charge transfer resistance, Qc is the Zn-Al diffusion layer capacitance, and Qct is the electric double layer equivalent capacitance between the Zn-Al diffusion layer and the substrate.

The equivalent circuit fitting data of the Zn-Al diffusion layer is shown in Table 7. When the immersion time is 0–360 h, the Zn-Al diffusion layer capacitance value Qc in the corrosion solution increases gradually. At this stage, the Zn-Al diffusion layer is invaded by a corrosive medium, increasing the value of Qc. The microscopic fluctuation of the Zn-Al diffusion layer surface leads to a large or small deviation of the electric double layer capacitance Qct, so that the Qct value changes irregularly, that is, the dispersion phenomenon. When immersed for 0–24 h, the Rc value decreases gradually, and the Rct value decreases slightly, which is consistent with the radius variation of the capacitive reactance arc in the Nyquist diagram. Analyses of the diagram data reveal that the diffusion layer has a much larger resistance Rc than the solution resistance Rs. In other words, the penetration of the corrosion solution makes the Zn-Al diffusion layer resistance Rc decrease. As the immersion time continues to increase, the Rc and Rct values increase gradually. The Zn-Al diffusion layer reacts with the corrosive solution, which generates a large number of corrosion products on the surface or inside and accumulates continuously. The phenomenon of the above reaction reduces the porosity of the Zn-Al diffusion layer, and strengthens the self-sealing effect, so that it slows down the progress of the electrochemical reaction.

## 4. Conclusions

(1) The Zn-Al diffusion layer aided by mechanical energy is composed of mainly Al_2_O_3_ and Γ_1_ phase (Fe_11_Zn_40_) and δ_1_ phase (FeZn_6.67_, FeZn_8.87_, FeZn_10.98_) Zn-Fe alloy. The FeZn_10.98_ alloy is mainly located in the region near the Zn-Al diffusion layer surface, and the FeZn_6.67_ alloy is mainly located in the interface between the Zn-Al diffusion layer and the substrate. There is a transition zone between the substrate and the Zn-Al diffusion layer with a thickness of about 5 μm, and a carbon-rich layer exists in this zone. In the direction of perpendicular to the surface, the content of Zn reduces gradually, the content of Fe increases slowly, and abrupt changes occur in this transition zone.

(2) As the immersion time is prolonged, the corrosion products accumulate on the surface and form corrosion pits, which increase the surface roughness. At the same time, the corrosion products of the Zn-Al diffusion layer accumulate on the surface to fill the surface crack and prevent the erosion of the corrosive medium, thereby reducing the corrosion rate of the Zn-Al diffusion layer and exerting the effect of enhancing the corrosion resistance.

(3) The results of salt spray corrosion test show that the corrosion products of the Zn-Al diffusion layer surface are flocculent in the initial corrosion stage, and the corrosion products are mainly ZnO and Zn_5_(OH)_8_Cl_2_H_2_O. In the middle corrosion stage, the needle-like corrosion products appear on the Zn-Al diffusion layer surface, and new corrosion products are ZnAl_2_O_4_ and FeOCl. In the later corrosion stage, the agglomerated corrosion products appear, the ZnAl_2_O_4_ disappears in the corrosion products, and Zn(OH)_2_ and Al(OH)_3_ appear. The appearance of Zn_5_(OH)_8_Cl_2_H_2_O increases the compactness of the corrosion products layer and slows down the corrosion rate of the Zn-Al diffusion layer. FeOCl is unstable and reacts with OH^−^ to form FeO(OH) that accumulates on the surface, which further slows down the corrosion rate.

(4) The results of the Zn-Al diffusion layer electrochemical test show that as the immersion time is prolonged, the self-corrosion potential of the Zn-Al diffusion layer shifts gradually to the positive electrode after moving to the negative electrode. The self-corrosion current density first rises and then falls, the polarization resistance Rp increases, and the corrosion resistance of the Zn-Al diffusion layer increases gradually.

(5) The impedance modulus of low-frequency region decreases, and the radius of the capacitive reactance arc becomes smaller within 0–24 h. At this time, the corrosion solution invades the Zn-Al diffusion layer. The corrosion diffusion stage is 72–360 h, and the impedance modulus and the capacitive reactance arc radius of the low-frequency region increases gradually. In this stage, the corrosion rate of the Zn-Al diffusion layer gradually decreases. After immersing for 360 h, the Zn-Al diffusion layer still has excellent corrosion resistance.

## Figures and Tables

**Table 1:** Elemental composition of 45 steel (wt. %).

Element	C	Mn	Si	Cu	P	S	Cr	Ni	Fe
wt. %	0.42–0.50	0.5–0.8	0.17–0.37	≤0.25	≤0.035	≤0.035	≤0.25	≤0.3	rest

**Table 2:** Percentage of constituent elements of the Zn-Al diffusion layer in different positions.

Element	1	2	3	4
O	7.5	7.17	6.08	5.52
Zn	83.11	81.65	83.32	80.98
Al	1.07	1.26	0.66	0.88
Fe	8.32	9.92	9.94	12.62

**Table 3:** Percentage of constituent elements in the region near the interface of the Zn-Al layer.

Element	1	2	3	4	5
C	––	––	51.85	47.12	47.78
Fe	12.80	13.86	7.94	7.70	13.12
Zn	87.20	86.14	40.22	45.18	39.10

**Table 4:** Main elemental weight ratio and atomic ratio of the Zn-Al diffusion layer for 360 h immersion.

Element	Weight Ratio (%)	Atomic Ratio (%)
O	29.15	59.85
Al	0.67	0.81
Cl	9.29	8.60
Fe	1.55	0.91
Zn	59.35	29.82

**Table 5:** Polarization curve fitting data of the Zn-Al diffusion layer.

Time	E (V)	Icorr (A/cm^2^)	βa (mV)	βc (mV)	Rp (Ω/cm^2^)
0.5 h	−0.9440	2.77 × 10^−6^	113.41	130.35	9124.1
10 h	−0.9369	2.84 × 10^−6^	79.651	366.16	9779.4
1 d	−0.9310	2.70 × 10^−6^	162.71	326.42	15,522
3 d	−0.9110	1.03 × 10^−6^	148.74	453.58	46,121
7 d	−0.8990	8.09 × 10^−7^	136.29	607.70	60,865
15 d	−0.8563	8.89 × 10^−7^	152.31	547.2	68,548

**Table 6:** The impedance modulus and corrosion rate of different immersion time of the Zn-Al diffusion layer.

Time (h)	0.5	10	24	72	168	360
Impedance modulus (Ω·cm^2^)	2378.6	1954.2	2024.8	3649.6	4976.9	8267.7
Corrosion rate (A/(cm^2^·h))	2.74 × 10^−6^	2.76 × 10^−6^	2.89 × 10^−6^	1.01 × 10^−6^	7.55 × 10^−7^	7.18 × 10^−7^

**Table 7:** Fitting data of the equivalent circuit of the Zn-Al diffusion layer.

Time (h)	0.5	10	24	72	168	360
Rs (Ω·cm^2^)	9.738	8.945	8.715	9.728	10.2	10.73
Qc (10^−4^ F·cm^−2^)	0.463	0.786	1.913	3.691	6.799	12.12
Rc (kΩ·cm^2^)	1.582	1.553	1.338	2.431	2.156	2.901
Qct (10^−4^ F·cm^−2^)	2.166	4.582	2.934	4.332	2.165	13.53
Rct (kΩ·cm^2^)	1.929	1.637	1.799	3.652	5.542	7.239
